# Racial and ethnic disparities in the uptake of SGLT2is and GLP-1RAs among Medicare beneficiaries with type 2 diabetes and heart failure, atherosclerotic cardiovascular disease and chronic kidney disease, 2013–2019

**DOI:** 10.1007/s00125-024-06321-2

**Published:** 2024-11-08

**Authors:** Eric Wang, Elisabetta Patorno, Farzin Khosrow-Khavar, Stephen Crystal, Chintan V. Dave

**Affiliations:** 1 Center for Pharmacoepidemiology and Treatment Science, Institute for Health, Health Care Policy and Aging Research, Rutgers University, New Brunswick, NJ USA; 2 Division of Pharmacoepidemiology and Pharmacoeconomics, Department of Medicine, Brigham and Women’s Hospital, Harvard Medical School, Boston, MA USA; 3 Department of Biostatistics and Epidemiology, Rutgers School of Public Health, Piscataway, NJ USA; 4 Center for Health Services Research, Institute for Health, Health Care Policy and Aging Research, Rutgers University, New Brunswick, NJ USA; 5 Rutgers School of Social Work, Rutgers University, New Brunswick, NJ USA; 6 Department of Pharmacy Practice and Administration, Ernest Mario School of Pharmacy, Rutgers University, Piscataway, NJ USA

**Keywords:** Chronic kidney disease, CVD, Diabetes, Disparities, GLP-1 receptor agonist, Heart failure, Medicare, Older people, SGLT2 inhibitor

## Abstract

**Aims/hypothesis:**

The aim of this study was to investigate racial and ethnic disparities in the use of sodium–glucose cotransporter 2 inhibitors (SGLT2is) and glucagon-like peptide-1 receptor antagonists (GLP-1RAs) among older adults with type 2 diabetes and cardiorenal conditions.

**Methods:**

Using Medicare fee-for-service data (2013–2019), this retrospective cohort study identified older adults (≥65 years) with type 2 diabetes initiating second-line therapies (SGLT2is, GLP1-RAs, dipeptidyl peptidase-4 inhibitors [DPP4is] and sulfonylureas [SUs]) with (1) heart failure (HF), (2) atherosclerotic cardiovascular disease (ASCVD), (3) chronic kidney disease (CKD) and (4) no recorded cardiorenal conditions. Participants were classified as non-Hispanic White, non-Hispanic Black and Hispanic. Multinomial regressions, adjusting for sociodemographic, clinical and county-level characteristics, were used to model the odds of initiating SGLT2is or GLP-1RAs within each cohort.

**Results:**

Black participants with HF, ASCVD, CKD or no recorded cardiorenal conditions had 35% (adjusted OR 0.65 [95% CI 0.61, 0.68]), 33% (0.67 [0.64, 0.69]), 32% (0.68 [0.64, 0.72]) and 24% (0.76 [0.74, 0.79]) lower odds of initiating SGLT2is, respectively, than White participants. Disparities ameliorated from 50–60% lower odds in 2013 to 17–18% in 2019. Similar patterns were observed for GLP-1RA uptake among Black participants. By contrast, Hispanic participants had similar odds of SGLT2i initiation in the HF and CKD cohorts as White participants, but 6% (0.94 [0.91, 0.98]) lower odds in the ASCVD cohort. Notable disparities for Hispanic participants compared with White participants were observed for GLP-1RA uptake in the HF, ASCVD, CKD and no cardiorenal conditions cohorts: 11% (0.89 [0.84, 0.94]), 16% (0.84 [0.81, 0.87]), 16% (0.84 [0.80, 0.89]) and 25% (0.75 [0.72, 0.78]) lower odds, respectively. Participants had greater odds than White participants of initiating DPP4is, which confer no cardiorenal benefits, across all cohorts (HF 1.25 [1.19, 1.31]; ASCVD 1.36 [1.32, 1.40]; CKD 1.32 [1.26, 1.38). Adjustment for social determinants of health did not meaningfully change the study findings.

**Conclusions/interpretation:**

Compared with White participants, disparities in the uptake of SGLT2is were evident for Black participants, and in the uptake of GLP-1RAs for both Black and Hispanic participants. This study highlights how type 2 diabetes management is evolving, while underscoring historical imbalances that have shown signs of abatement.

**Graphical Abstract:**

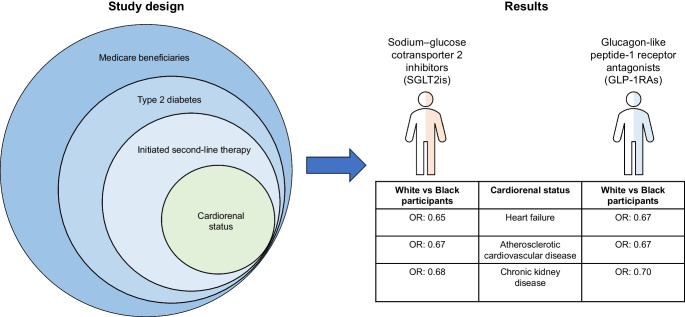

**Supplementary Information:**

The online version of this article (10.1007/s00125-024-06321-2) contains peer-reviewed but unedited supplementary material.


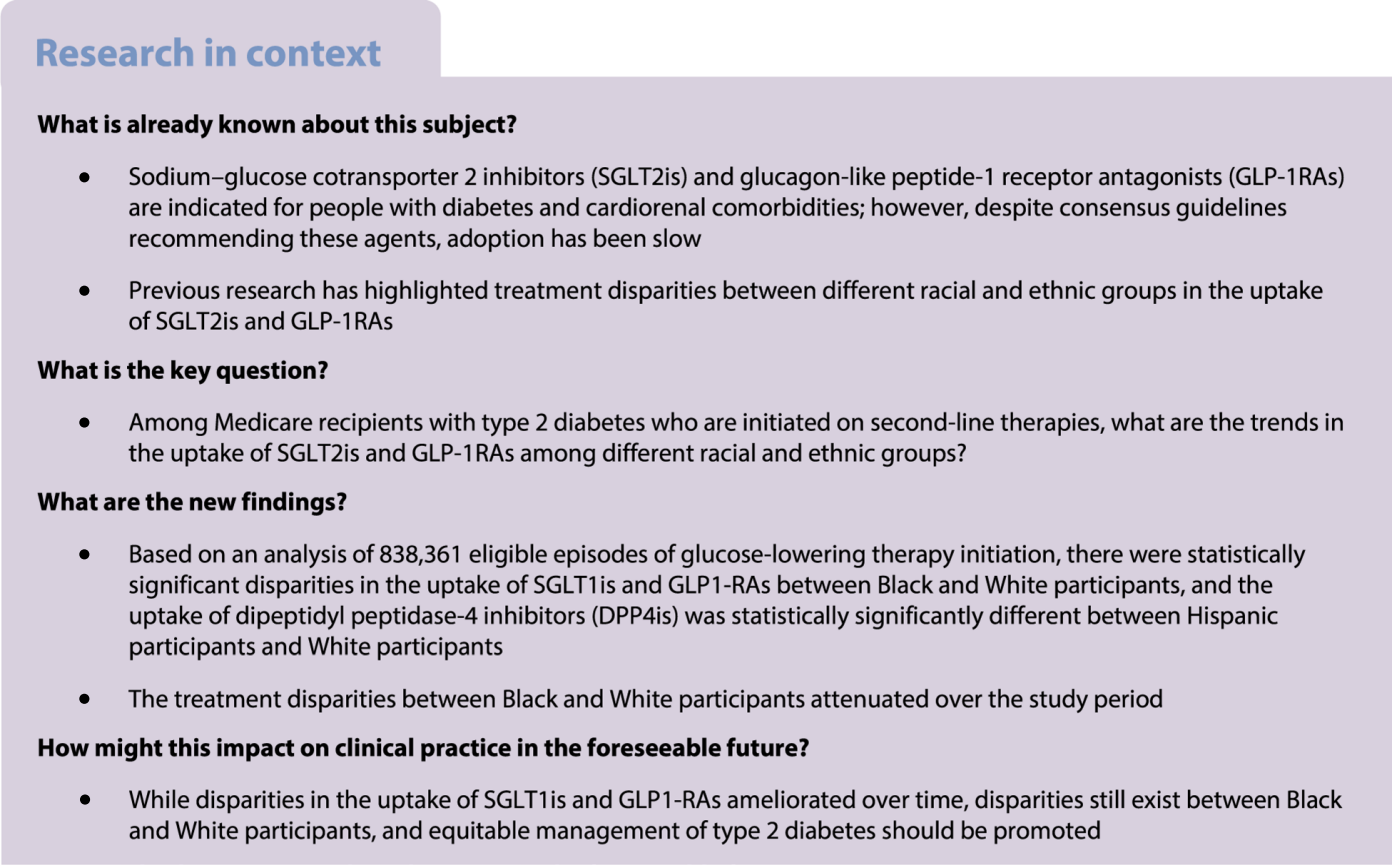


## Introduction

Because of the shared pathophysiological mechanisms underpinning the development of type 2 diabetes and cardiorenal conditions, coupled with the micro- and macrovascular consequences of chronic unmitigated hyperglycaemia, more than 35% of individuals with type 2 diabetes are co-diagnosed with atherosclerotic cardiovascular disease (ASCVD), congestive heart failure (HF) or chronic kidney disease (CKD) [1, 2]. Compared with diabetes alone, the co-occurrence of cardiorenal conditions augurs a clinical course characterised by accelerated disease progression, excess healthcare spending and increased risk of all-cause mortality [3, 4].

In recent years, sodium–glucose cotransporter 2 inhibitors (SGLT2is) and glucagon-like peptide-1 receptor antagonists (GLP-1RAs) have demonstrated benefits in preventing deleterious cardiorenal endpoints among individuals with type 2 diabetes, shifting disease management from a glucocentric approach to one that prioritises long-term cardiorenal health [5–7]. These accumulating findings have led to corresponding changes in medication package inserts, and updates to national and international guidelines. Currently, these guidelines recommend SGLT2is and GLP-1RAs as the preferred therapy among individuals with type 2 diabetes and comorbid ASCVD or HF, and SGLT2is among those with comorbid CKD [8, 9].

Despite consensus recommendations and accumulating clinical evidence, the adoption of SGLT2is and GLP-1RAs has been slow in those with type 2 diabetes and comorbid cardiorenal conditions [10, 11]. Previous research has highlighted a pattern of treatment disparities that is consistent across various data sources [12–17]. However, trends in the uptake of SGLT2is and GLP-1RAs within the Medicare population, factoring in race and ethnicity and community-level social determinants of health, remain relatively understudied. Given that racial and ethnic minority groups experience higher rates of type 2 diabetes-related morbidity and mortality, examining these disparities is important [18]. Hence, our study aimed to assess the adoption of these therapies among older individuals with type 2 diabetes and comorbid ASCVD, HF or CKD and in those with type 2 diabetes without cardiorenal conditions, and to quantify the secular trends in uptake of these agents by race and ethnicity.

## Methods

This study was approved by the Rutgers University Institutional Review Board and the requirement for informed consent was waived.

### Data source and study population

Study participants were drawn from insurance claims to Medicare, a US federal programme that provides healthcare to US citizens aged ≥65 years, from 2013 (coinciding with the availability of SGLT2is) to 2019. This dataset provides information on a highly representative sample of older US adults as a result of Medicare's near-universal coverage.

More specifically, we used a 50% random sample of Medicare fee-for-service beneficiaries enrolled in Part D. The Medicare fee-for-service database provides patient-level information on pharmacy and healthcare enrolment status; sociodemographic variables including date of birth, biological sex, and race and ethnicity; inpatient and outpatient services rendered (ICD-9 and ICD-10, Current Procedural Terminology codes, 4th edn); and outpatient pharmacy dispensing (drug name, date of dispensing, and number of days’ supply).

Cohort membership was restricted to those aged ≥65 years diagnosed with type 2 diabetes and with no evidence of end-stage kidney disease or cancer and who were continuously enrolled in the database for at least 1 year prior to medication initiation. Individuals were included if they had initiated one or more of the following four second-line glucose-lowering medications: SGLT2is, GLP1-RAs, dipeptidyl peptidase-4 inhibitors (DPP4is) or sulfonylureas (SUs). Treatment initiation was defined as a filled prescription for a study medication preceded by non-use of the study medication during the baseline period (i.e. 365 days before treatment initiation). Participants were allowed to contribute more than one treatment initiation episode as long as the eligibility criteria were met at the time of initiation. Our rationale for focusing on individuals newly initiating the glucose-lowering medications of interest was driven by three factors. First, by including only new users, we were able to focus on a period when a treatment change was being planned, offering an opportunity for consideration of a more guideline-concordant treatment. Second, this approach allowed us to clearly define an index date (i.e. date of medication initiation), enabling an assessment of participant characteristics prior to treatment initiation. Finally, such a design is more sensitive to shifts in prescribing patterns, as it focuses on individuals initiating new treatments rather than those maintaining long-term, ongoing therapies.

### Participant characteristics

To describe the study population, we examined participant characteristics such as age, sex (available from the Social Security Administration [SSA] files) and selected comorbid conditions (e.g. atrial fibrillation, hypertension and hyperlipidaemia). All participant characteristics, including the eligibility criteria, were assessed during the baseline period (i.e. 365 days prior to medication initiation). Participants with type 2 diabetes were classified into four non-mutually exclusive cohorts with (1) HF, (2) ASCVD, (3) CKD and (4) no cardiorenal conditions recorded in Medicare insurance claims.

Using the Research Triangle Institute (RTI) modified race and ethnicity variable [19] (available from SSA enrolment data), we categorised study participants into four groups: non-Hispanic White adults (‘White’), non-Hispanic Black adults (‘Black’), Hispanic adults and ‘other race and ethnicity’. The ‘other race and ethnicity’ category included American Indian/Alaska Native adults, Asian American/Pacific Islander adults and individuals missing race information, accounting for 6.5% of our cohort, precluding more granular assessments for these groups. Differences by sex and gender were not considered in the study design as the treatments are not differentiated by sex and gender. Additionally, only data on biological sex were available for analysis.

Using participants’ county of residence at the time of glucose-lowering medication initiation, we linked participant records to county-level Social Vulnerability Index (SVI) data for 2010, 2014, 2016 and 2018. Briefly, the SVI reports on the relative vulnerability of each US county through 15 social factors organised into four domains corresponding to: (1) socioeconomic status; (2) household composition and disability; (3) minority status and language; and (4) housing type and transportation. Counties are ranked according to a summary score, expressed as percentiles ranging from 0 (least vulnerable) to 1 (most vulnerable).

### Statistical analysis

Within the study cohort of older Medicare beneficiaries initiating the four second-line glucose-lowering therapies of interest, we divided the study period from 2013 to 2019 into seven calendar year intervals, and medication initiation episodes were assigned to these intervals based on their index date. All analyses were stratified according to the four cohorts of (1) HF, (2) ASCVD, (3) CKD and (4) no cardiorenal conditions. For each calendar year, we described the proportion of initiations attributable to either SGLT2is, GLP-1RAs, DPP4is or SUs (numerator) relative to the total initiations across all four drug classes (denominator). A time trend analysis was performed to examine the presence of linear secular trends over the 7 year period.

A multinomial logistic regression was employed that modelled the choice of drug class received (i.e. SGLT2is, GLP-1RAs, SUs, DPP4is) as the dependent variable and race and ethnicity as the independent variable of interest, while adjusting for the association of age, sex, overall SVI and the four domains of the SVI (socioeconomic status; household composition and disability; minority status and language; and housing type and transportation), other clinical characteristics such as diabetic neuropathy, retinopathy and nephropathy, baseline use of medications such as metformin, insulin or other glucose-lowering therapies, and baseline comorbid conditions such as hypertension, hyperlipidaemia, atrial fibrillation, obesity and liver cirrhosis. In these models, SUs were selected as the reference category because of their long-established availability, generic status, lower cost relative to SGLT2is, GLP-1RAs and DPP4is, and lack of cardiorenal benefit. This choice enabled a more clinically relevant comparison across the three relatively newer, branded therapies. These models were estimated for each of the four cohorts: (1) HF, (2) ASCVD, (3) CKD and (4) no cardiorenal conditions.

We conducted three additional analyses. First, we estimated adjusted models for each calendar year of the study, providing a detailed look at the adjusted trends in any racial and ethnic disparities. Second, we estimated an unadjusted model focusing exclusively on race and ethnicity, to provide a foundational understanding of how the adoption of newer glucose-lowering medications varies across racial and ethnic groups without consideration of other variables. Finally, to assess the effect on our study estimates of the inclusion of multiple drug initiation episodes, we conducted a sensitivity analysis restricting the study cohort to the first initiation episode.

## Results

**Fig. 1:**
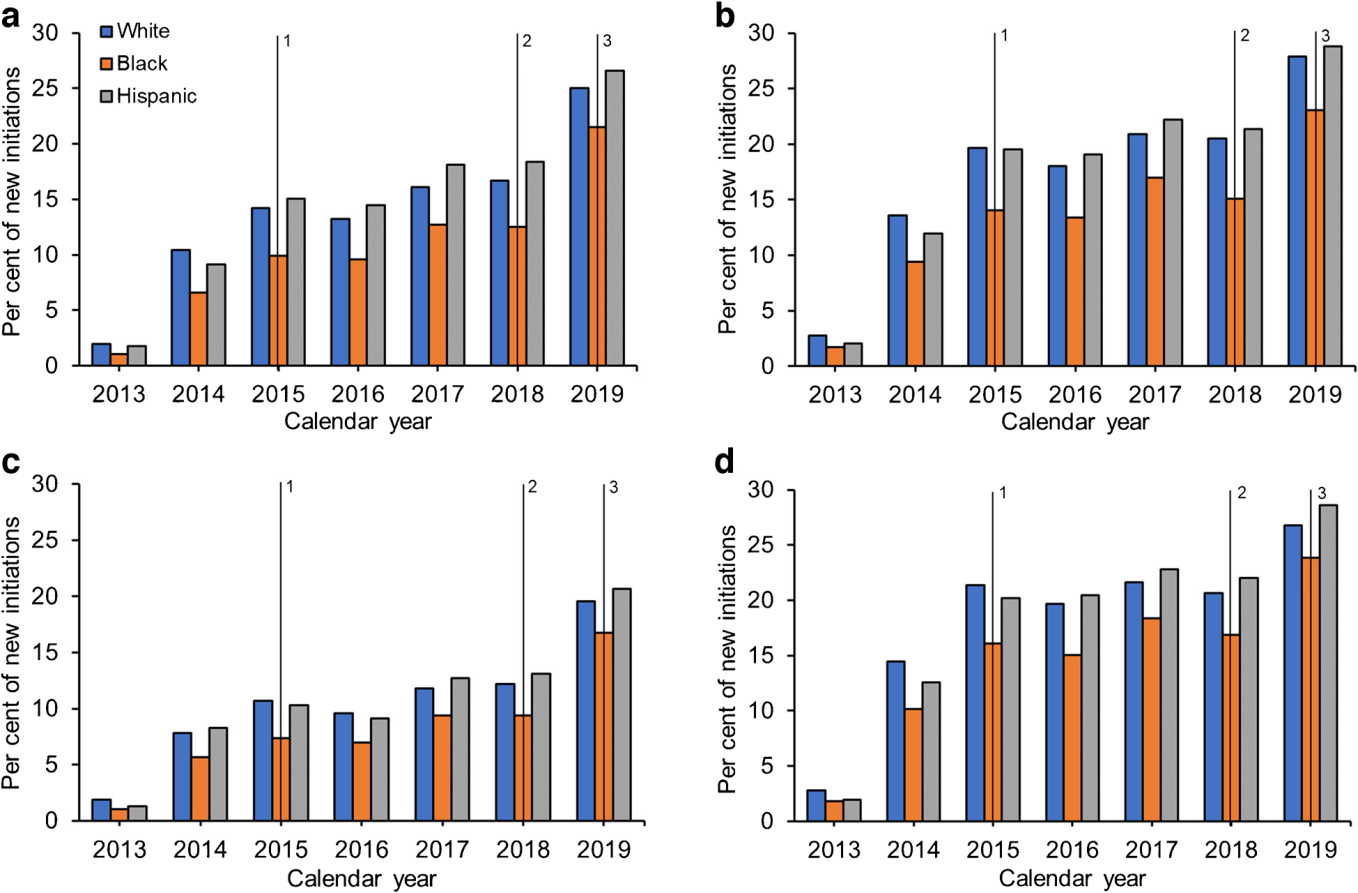
Temporal patterns (2013–2019) in the proportions of new initiations of SGLT2is by race and ethnicity and cardiorenal condition: (**a**) comorbid HF; (**b**) comorbid ASCVD; (**c**) comorbid CKD; (**d**) no comorbid cardiorenal conditions. 1: EMPA-REG OUTCOME trial results published, highlighting the cardiovascular benefits of SGLT2is [6]. 2: ADA guideline shifts, indicating SGLT2is and GLP1-RAs as preferred therapies for individuals with type 2 diabetes and comorbid cardiovascular conditions [36]. 3: CREDENCE trial results published, highlighting SGLT2i benefits in CKD [37]

We identified 1,115,332 potential new users of the study medications from a total of 58,874,099 prescription fills in the Medicare database between 2013 and 2019. Individuals with prior use of the study drugs during the baseline period (*n*=111,231), a type 1 diabetes diagnosis (*n*=162,810) and missing county data for SVI linkage (*n*=2930) (electronic supplementary material [ESM] Fig. 1) were excluded. After applying these criteria, the final study cohort consisted of 838,361 eligible episodes of glucose-lowering therapy initiation, of which 152,214, 412,890, 154,849 and 350,707 episodes had evidence of HF, ASCVD, CKD and no cardiorenal conditions, respectively. Compared with White participants, Black participants were slightly younger and less likely to be male (Table 1; see ESM Table 1 for stratified results by race and ethnicity and cardiorenal condition status and ESM Table 2 for stratified results by cardiorenal condition status). Black and Hispanic participants had a higher prevalence of diabetes-related complications and CKD than White participants.

**Table 1:** Baseline clinical characteristics for participants with type 2 diabetes initiating second-line glucose-lowering therapies by race and ethnicity, 2013–2019

Characteristic	White (*n*=619,825)	Black (*n*=75,570)	Hispanic (*n*=77,515)
Age, mean (SD), years	74.4 (6.6)	73.7 (6.6)	74.2 (6.6)
Male	306,046 (49.4)	26,030 (34.4)	31,905 (41.2)
SVI percentile			
SVI (overall), median (IQR)	51.5 (41.9)	68.1 (32.4)	75.4 (34.9)
Baseline complications			
Diabetic neuropathy	158,449 (25.6)	21,160 (28.0)	22,734 (29.3)
Diabetic retinopathy	72,367 (11.7)	12,699 (16.8)	15,311 (19.8)
Diabetic nephropathy	48,432 (7.8)	7826 (10.4)	8862 (11.4)
Baseline comorbid medical conditions			
CKD	111,090 (17.9)	17,583 (23.3)	14,232 (18.4)
ASCVD	310,924 (50.2)	35,954 (47.6)	36,594 (47.2)
HF	113,728 (18.3)	16,076 (21.3)	13,116 (16.9)
Hypertension	570,296 (92.0)	72,990 (96.6)	71,885 (92.7)
Hyperlipidaemia	543,357 (87.7)	63,481 (84.0)	65,952 (85.1)
Atrial fibrillation	112,142 (18.1)	7551 (10.0)	7624 (9.8)
Obesity	194,937 (31.5)	23,466 (31.1)	21,314 (27.5)
Liver cirrhosis	8940 (1.4)	782 (1.0)	1680 (2.2)
Drug initiation over study period			
SGLT2i	119,627 (19.3)	11,529 (15.3)	14,803 (19.1)
GLP1-RA	114,942 (18.5)	12,175 (16.1)	12,325 (15.9)
DPP4i	111,887 (18.1)	18,830 (24.9)	21,361 (27.6)
SU	273,369 (44.1)	33,036 (43.7)	29,026 (37.4)

Data are *n* (%) unless indicated otherwise

### Overall medication initiation trends by race and ethnicity

Over the study period, SGLT2i initiation increased notably among White participants with HF (23.1% increase from 2013 to 2019, *p*=0.003 for trend), ASCVD (25.1%, *p*=0.007), CKD (17.7%, *p*=0.004) and no cardiorenal conditions (23.9%, *p*=0.02) (Fig. 1; ESM Table 3). Conversely, among Black participants, these increases were less pronounced, with SGLT2i initiation increasing by 20.4% (*p*=0.002), 21.4% (*p*=0.004), 15.7% (*p*=0.004) and 22.0% (*p*=0.005) for the respective cohorts. Across the four cohorts, the disparity in SGLT2i use between Black and White participants was evident for all study years, emerging as early as 2013, intensifying annually and peaking in 2015–2016. Although signs of improvement emerged, this disparity persisted up to 2019. Initiation rates of SGLT2is were comparable between Hispanic and White participants.

**Fig. 2:**
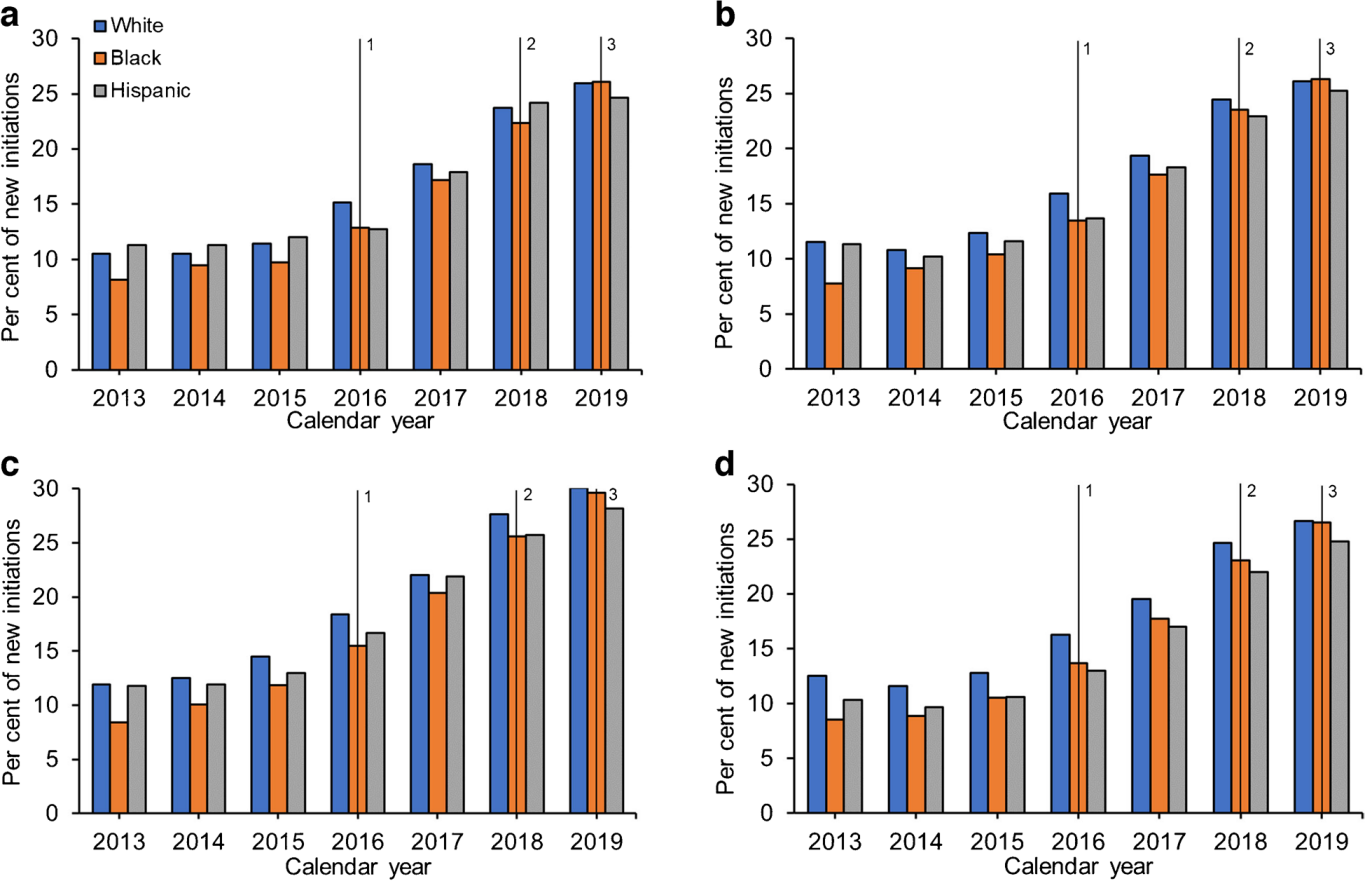
Temporal patterns (2013–2019) in the proportions of new initiations of GLP1 RAs by race and ethnicity and cardiorenal condition: (**a**) comorbid HF; (**b**) comorbid ASCVD; (**c**) comorbid CKD; (**d**) no comorbid cardiorenal conditions. 1: LEADER and SUSTAIN-6 trial results published, highlighting the cardiovascular benefits of GLP1-RAs [38, 39]. 2: ADA guideline shifts, indicating SGLT2is and GLP1-RAs as preferred therapies for individuals with type 2 diabetes and comorbid cardiovascular conditions [36]. 3: REWIND trial results published, highlighting the cardiovascular benefits of GLP-1RAs [40]

In contrast to the unadjusted patterns observed for SGLT2is, the unadjusted disparities in GLP-1RA use were less pronounced. Among White participants, GLP-1RA use increased by 15.5% (*p*<0.001 for trend), 14.5% (*p*<0.001), 18.2% (*p*<0.001) and 14.1% (*p*<0.001) among those with HF, ASCVD, CKD and no cardiorenal conditions, respectively (Fig. 2; ESM Table 4). Meanwhile, among Black participants, corresponding increases of 17.9% (*p*<0.001), 18.6% (*p*<0.001), 21.2% (*p*<0.001) and 17.9% (*p*<0.001) were seen. While some evidence of disparities in GLP-1RA uptake between White and Black participants was observed in the earlier study years, these disparities diminished around 2016 and were no longer apparent by the end of the study period in 2019.

Although the use of DPP4is and SUs declined in all four cardiorenal cohorts over the study period, the decline in DPP4i use was less pronounced among Black and Hispanic participants (ESM Figs 2 and 3; ESM Tables 5 and 6).

### Adjusted ORs for medication initiation for Black vs White participants

**Fig. 3:**
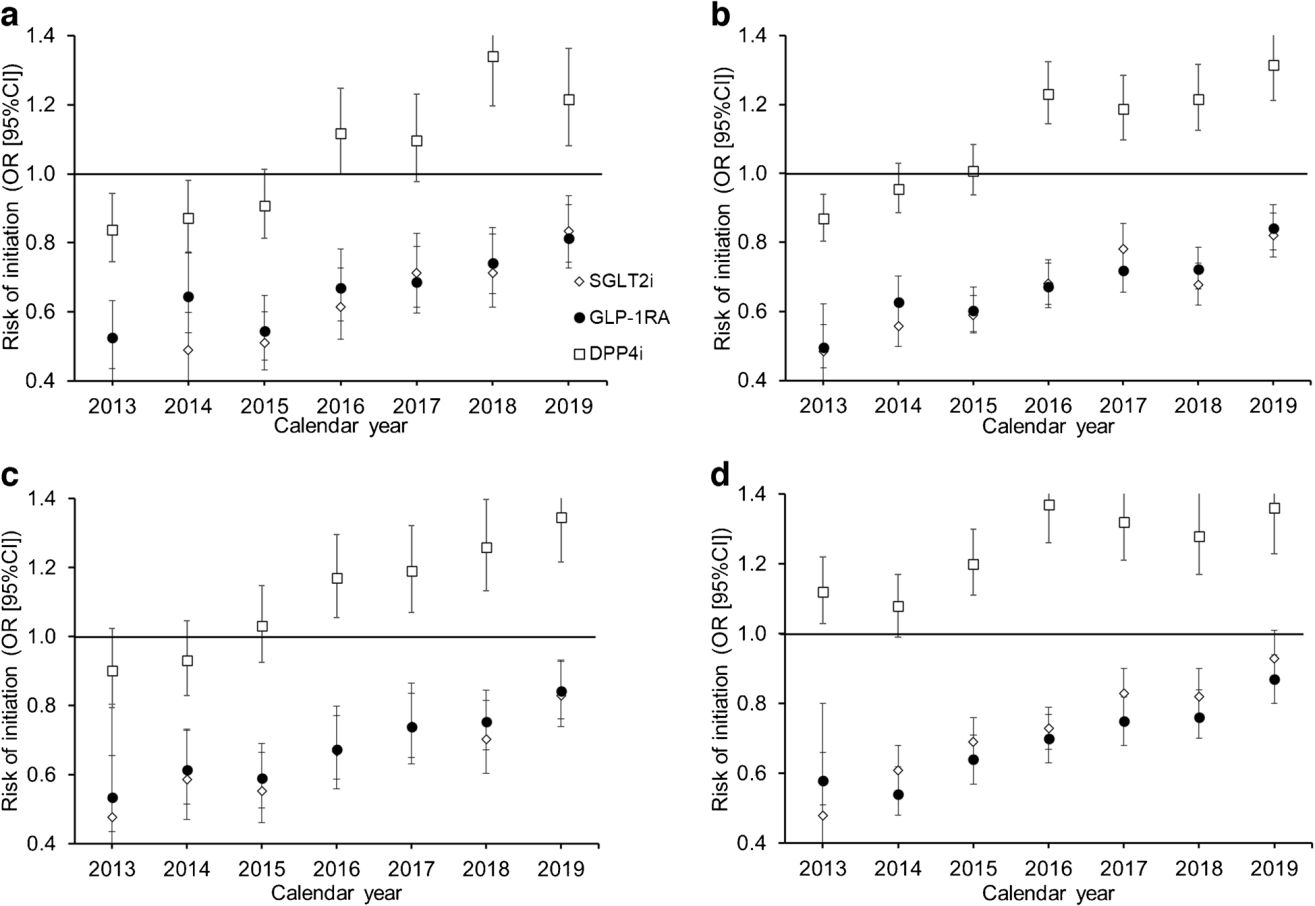
Adjusted multinomial regression models of the odds of initiating second-line therapies per year by comorbid cardiorenal condition and drug class for Black vs White participants, with SUs as the reference group: (**a**) comorbid HF; (**b**) comorbid ASCVD; (**c**) comorbid CKD; (**d**) no comorbid cardiorenal conditions

After adjusting for the association of age, sex, SVI and other clinical characteristics, and using SUs as the reference category, Black participants with HF, ASCVD, CKD and no cardiorenal conditions had 35% (OR 0.65, 95% CI 0.61, 0.68), 33% (OR 0.67, 95% CI 0.64, 0.69), 32% (OR 0.68, 95% CI 0.64, 0.72) and 24% (OR 0.76, 95% CI 0.74, 0.79) lower odds of initiating SGLT2is, respectively, than White participants over the full 2013–2019 period (Fig. 3; ESM Table 7). The likelihood of DPP4i initiation increased over the study period and Black participants were more likely than White participants to initiate DPP4is regardless of comorbid cardiorenal condition.

The disparities in SGLT2i initiation were most prominent in the earlier study years prior to 2016 for all four cohorts, with Black participants having a 50–60% lower odds of initiation than White participants. However, these differences attenuated in a linear fashion year-on-year, although they did not dissipate entirely. By 2019, Black participants with HF, ASCVD and CKD still had 17% (OR 0.83, 95% CI 0.74, 0.94), 18% (OR 0.82, 95% CI 0.76, 0.89) and 17% (OR 0.83, 95% CI 0.74, 0.93) lower odds of SGLT2i initiation, respectively, than White participants (ESM Table 8).

While disparities for SGLT2is were apparent in both unadjusted (Fig. 1) and adjusted (Fig. 3) analyses, the disparities in GLP-1RA initiation for Black compared with White participants were not as apparent in unadjusted analyses (Fig. 2) but became evident after adjusting for relevant clinical characteristics (Fig. 3). Black participants with HF, ASCVD, CKD and no cardiorenal conditions had 33% (OR 0.67, 95% CI 0.63, 0.70), 33% (OR 0.67, 95% CI 0.65, 0.70), 30% (OR 0.70, 95% CI 0.66, 0.73) and 29% (OR 0.71, 95% CI 0.68, 0.73) lower odds of GLP-1RA initiation, respectively, than White participants over the full study period (Fig. 3; ESM Table 7). Similar to SGLT2i initiation, there appeared to be a reduction in the observed disparities over the course of the study period (HF: 2013 OR 0.53 vs 2019 OR 0.81; ASCVD: 2013 OR 0.50 vs 2019 OR 0.84; CKD: 2013 OR 0.54 vs 2019 OR 0.84).

In the sensitivity analyses adjusting only for age and sex, the observed disparities were less pronounced (ESM Tables 9 and 10), indicating that the additional adjustments for clinical and other characteristics in the fully adjusted models resulted in the increased disparities observed. In the sensitivity analyses restricting the cohorts to the first initiation episode, study findings were similar to the finding of the primary analysis (ESM Table 11).

### Adjusted ORs for medication initiation for Hispanic vs White participants

**Fig. 4:**
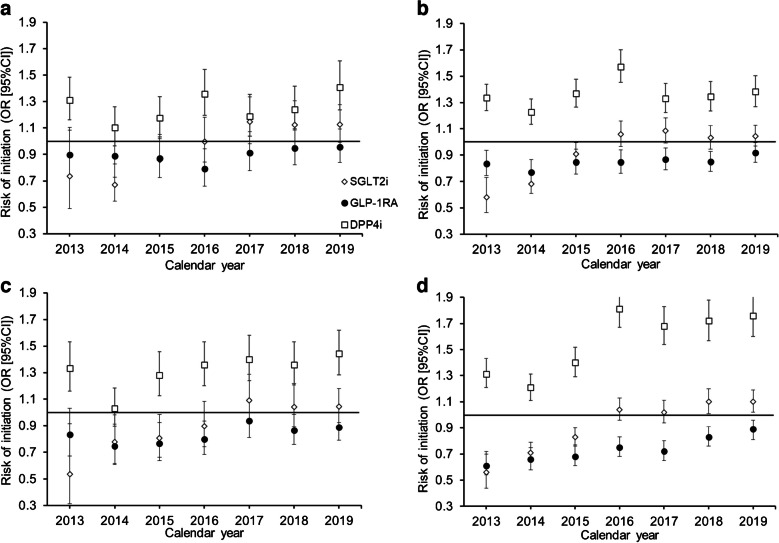
Adjusted multinomial regression models of the odds of initiating second-line therapies per year by comorbid cardiorenal condition and drug class for Hispanic vs White participants, with SUs as the reference group: (**a**) comorbid HF; (**b**) comorbid ASCVD; (**c**) comorbid CKD; (**d**) no comorbid cardiorenal conditions

Disparities in the initiation of second-line therapies were less apparent for Hispanic participants. Overall, Hispanic participants with HF and CKD had similar odds of SGLT2i initiation as White participants (HF: OR 0.99, 95% CI 0.93, 1.05; CKD: OR 0.95, 95% CI 0.89, 1.02), while those with ASCVD and no cardiorenal conditions had 6% (OR 0.94, 95% CI 0.91, 0.98) and 7% (OR 0.93, 95% CI 0.90, 0.96) lower odds of initiation, respectively, than White participants (Fig. 4; ESM Table 7). In contrast, there appeared to be greater disparities in the uptake of GLP-1RAs by Hispanic participants compared with White participants. Hispanic participants with HF, ASCVD, CKD and no cardiorenal conditions had 11% (OR 0.89, 95% CI 0.84, 0.94), 16% (OR 0.84, 95% CI 0.81, 0.87), 16% (OR 0.84, 95% CI 0.80, 0.89) and 25% (OR 0.75, 95% CI 0.72, 0.78) lower odds of initiation of GLP-1RAs, respectively. Hispanic participants were more likely than White participants to initiate DPP4is across the study period regardless of comorbid cardiorenal condition (HF: OR 1.25, 95% CI 1.19, 1.31; ASCVD: OR 1.36, 95% CI 1.32, 1.40; CKD: OR 1.32, 95% CI 1.26, 1.38).

In the fully adjusted model by year, trends over time were not as clear as in the analysis comparing Black participants with White participants. The likelihood of DPP4i initiation increased over the study period and Hispanic participants were more likely than White participants to initiate DPP4is regardless of comorbid cardiorenal condition (ESM Table 12).

In the sensitivity analyses adjusting only for age and sex, Hispanic participants with comorbid cardiorenal conditions were more likely to initiate SGLT2is and GLP1-RAs than White participants with comorbid cardiorenal conditions (ESM Table 9). Trends across years when adjusting only for age and sex did not show much disparity between Hispanic participants and White participants in the initiation of SGLT2is and GLP1-RAs and, in later years, seemed to favour Hispanic participants (ESM Table 13). In the sensitivity analysis restricting the cohort to the first initiation episode only, the study findings were similar to the findings of the primary analysis (ESM Table 11).

## Discussion

Racial and ethnic disparities among second-line therapy initiators, while previously postulated, have not been comprehensively studied in Medicare data. We analysed Medicare records from 2013 to 2019 for beneficiaries aged ≥65 years with a diagnosis of type 2 diabetes who initiated second-line therapy. Multinomial regression models were used to estimate the probability of initiating SGLT2is, GLP-1RAs or DPP4is, with SUs as the reference category. Our investigation revealed several notable insights, shedding light on these inequities. First, despite having a higher burden of cardiorenal conditions, Black participants had a lower likelihood of SGLT2i and GLP-1RA initiation than White participants, both before and after adjusting for covariates. In the fully adjusted model, these disparities were most prominent in the early study years, but ameliorated over time, decreasing for SGLT2is from a 50–60% initiation gap in 2013 to a 17–18% gap in 2019. Second, Hispanic participants exhibited lower SGLT2i use than White participants in the early years, but this disparity rapidly diminished. Conversely, for GLP-1RAs, the disparities with White participants endured, with a consistent 8–11% lower adjusted likelihood of initiation of GLP-1RAs in 2019 for the ASCVD and CKD cohorts. Third, both Black and Hispanic participants exhibited higher rates of DPP4i initiation than White participants. Lastly, while there was evidence of some variability in uptake by cardiorenal condition, the observed disparities were similar in magnitude across the four subgroups.

Our analysis revealed statistically significantly lower rates of SGLT2i and GLP-1RA initiation among Black than White participants and, to a lesser extent, among Hispanic participants. Differences in initiation of SGLT2is between Black and White participants persisted despite adjusting for clinical factors and community-level vulnerability, suggesting the involvement of additional determinants. Interestingly, social determinants of health do not appear to explain the differences in uptake between racial and ethnic groups, as adjustment for this did not meaningfully change the results. Cost considerations, attributed to SGLT2is and GLP-1RAs still being branded products, could be one potential explanation contributing to the disparities observed. However, it is notable that, despite cost considerations, DPP4is, another branded product that confers no cardiorenal benefits, had higher initiation rates among Black and Hispanic participants than White participants. For GLP1-RAs, adjusting for covariates allowed for clarification of the differential initiation patterns between Black and White participants.

Another potential influence on these disparities is prescribing inertia [20], as cardiovascular outcomes trials and guideline updates related to SGLT2is and GLP-1RAs are relatively recent and initiation of these newer medications may be limited to early adopters. The specialty of the prescribing physician may also have played a role, as there are data indicating higher rates of adoption of newer glucose-lowering therapies among certain medical specialties (e.g. endocrinologists) [12]. Lower rates of access to specialists among Black and Hispanic participants may have contributed to the disparities observed in the earlier years [21]. Further, the unique adverse reaction profiles of these newer medications, including diabetic ketoacidosis, urogenital infections and gastrointestinal side effects [6, 22–25], may have led to provider and patient hesitancy. Additionally, cultural factors and barriers such as strong adherence to cultural norms and religious beliefs, linguistic diversity, low health literacy levels and low levels of accessibility to culturally appropriate services/information may also influence the acceptability and adoption of newer glucose-lowering medications [26]. Finally, implicit biases in medical decision-making processes, disparate perceptions of healthcare systems or structural racism may also collectively contribute to the observed disparities in adopting newer therapies [27, 28].

Although data on SGLT2i and GLP-1RA disparities in older Medicare patients with type 2 diabetes and comorbid HF, ASCVD and CKD are lacking, our findings generally align with existing literature on the diffusion of newer glucose-lowering therapies and racial and ethnic disparities [11–13, 16, 29]. Previous studies have demonstrated that Black populations are less likely to initiate newer second-line therapies [12, 29, 30], a trend that is consistent with broader findings that racial and ethnic minority groups receive suboptimal diabetes care [31, 32]. This discrepancy persists even in healthcare systems with equal medication access, such as the Veterans Health Administration [14]. Additionally, uptake of these agents is low across all racial and ethnic groups for all eligible individuals regardless of setting [33, 34]. While our study results are consistent with previous findings, our study also contextualises trends in SGLT2i and GLP-1RA initiation against initiation of other second-line glucose-lowering therapies (including DPP4is and SUs), separately examines individuals co-diagnosed with HF, ASCVD and CKD, and, most notably, includes an in-depth exploration of unadjusted and adjusted temporal trends.

This study is characterised by several notable strengths. It leverages an extensive and highly representative dataset encompassing a geographically and racially diverse sample of older US Medicare beneficiaries aged ≥65 years with type 2 diabetes. It also systematically assesses the presence of racial and ethnic disparities in SGLT2i and GLP-1RA uptake across individual cardiorenal indications. Further, the research not only presents an overarching perspective spanning 2013–2019 but also delves into adjusted secular trends, shedding light on both the initial years of pronounced disparities followed by an encouraging attenuation of these differences. Finally, this study contextualises the adoption of SGLT2is and GLP-1RAs against the backdrop of the two other most commonly used second-line therapies: DPP4is and SUs.

There are also several study limitations. First, we used a 365 day washout window to define new medication initiation; however, it is possible that participants may have used the study medications prior to this baseline period. Second, information on social vulnerability was aggregated at county level and individual-level data were therefore unavailable. Third, the study findings are generalisable only to older individuals with type 2 diabetes with Medicare fee-for-service insurance. Fourth, we were unable to account for formulary differences, which may have contributed to some disparities. Fifth, data on diabetes duration, disease control and visits to specialists such as cardiologists, endocrinologists and nephrologists were not available. Finally, the study period ended in 2019 because of the lag time in data availability, preventing an examination of recent trends in response to 2019/2020 guideline changes (when SGLT2is and GLP1-RAs were recommended for those with established cardiorenal risks irrespective of glycaemic management) [35] and a comprehensive assessment of the impact of COVID-19 on disparities in the adoption of these newer therapies.

In conclusion, among older Medicare beneficiaries with type 2 diabetes initiating second-line glucose-lowering therapies, multinomial regression analysis using SUs as the reference revealed statistically significant disparities in the uptake of SGLT2is among Black participants and in the uptake of GLP-1RAs among both Black and Hispanic participants. This study highlights the historical patterns of differential diffusion of newer second-line therapies following market entry and gradual acceptance as standard of care in type 2 diabetes management, and underscores the historical racial and ethnic imbalances, which have shown encouraging signs of abatement. While our study noted an amelioration of disparities in the initiation of SGLT2is and GLP-1RAs over time, statistically significant differences remained at the end of the study. Such disparities in the use of these therapies may exacerbate existing inequalities in cardiorenal health and outcomes among racial and ethnic minority groups with diabetes. Understanding the reasons for these disparities in medication use is necessary to promote equitable management of type 2 diabetes and outcomes among older Black and Hispanic individuals in the USA, and to optimise equity of access to new treatments with documented population health benefits.

## Supplementary Information

Below is the link to the electronic supplementary material.

ESM (PDF 763 KB)

## Data Availability

The authors declare that the raw data used in the manuscript are not publicly available to share due to data user agreements in place with the Centers for Medicare and Medicaid Services (CMS). Raw data may be acquired individually through the CMS.

## Abbreviations

ASCVD: Atherosclerotic cardiovascular disease
CKD: Chronic kidney disease
DPP4i: Dipeptidyl peptidase-4 inhibitor
GLP-1RA: Glucagon-like peptide-1 receptor antagonist
HF: Heart failure
SGLT2i: Sodium–glucose cotransporter 2 inhibitor
SSA: Social Security Administration
SU: Sulfonylurea
SVI: Social Vulnerability Index

## Authors’ relationships and activities

EW is an employee of Daiichi Sankyo. CVD is the principal investigator on grants from the NHLBI (1R01HL163163) and JDRF (3-SRA-2022-1257-S-B) and reports consulting fees from the US Food and Drug Administration (FDA) and Takeda. EP has received research grants from the Patient-Centered Outcomes Research Institute (DB-2020C2-20326) and the US FDA (5U01FD007213) that are not related to the topic of this work. She is the principal investigator on a research grant to the Brigham and Women’s Hospital from Boehringer Ingelheim that is not related to the topic of this work.
