# Supplementary material for: Racial and ethnic disparities in the uptake of SGLT2is and GLP-1RAs among Medicare beneficiaries with type 2 diabetes and heart failure, atherosclerotic cardiovascular disease and chronic kidney disease, 2013–2019
Source: Diabetologia. 2024 Nov 8;68(1):94–104. doi: 10.1007/s00125-024-06321-2 (PMC11663158; doi:10.1007/s00125-024-06321-2)
Supplement: Supplementary file 1 — ESM (PDF 763 KB) [file 125_2024_6321_MOESM1_ESM.pdf]

Electronic Supplementary Material (ESM)

ESM Table 1: Clinical characteristics for T2DM patients initiating 2nd line glucose-lowering therapies by race/ethnicity from 2013-2019

**Total Population**			
	**White (n= 619,825)**	**Black (n= 75,570)**	**Hispanic (n=77,515)**
**Age, mean (SD), years**	74.4 (6.6)	73.7 (6.6)	74.2 (6.6)
**Male, n (%)**	306,046 (49.4)	26,030 (34.4)	31,905 (41.2)
**SVI Percentile**			
SVI (Overall), median (IQR)	51.5 (41.9)	68.1 (32.4)	75.4 (34.9)
**Baseline Complications, n (%)**			
Diabetic Neuropathy	158,449 (25.6)	21,160 (28.0)	22,734 (29.3)
Diabetic Retinopathy	72,367 (11.7)	12,699 (16.8)	15,311 (19.8)
Diabetic Nephropathy	48,432 (7.8)	7,826 (10.4)	8,862 (11.4)
**Baseline Comorbid Medical Conditions, n (%)**			
CKD	111,090 (17.9)	17,583 (23.3)	14,232 (18.4)
ASCVD	310,924 (50.2)	35,954 (47.6)	36,594 (47.2)
HF	113,728 (18.3)	16,076 (21.3)	13,116 (16.9)
Hypertension	570,296 (92.0)	72,990 (96.6)	71,885 (92.7)
Hyperlipidemia	543,357 (87.7)	63,481 (84.0)	65,952 (85.1)
Atrial Fibrillation	112,142 (18.1)	7,551 (10.0)	7,624 (9.8)
Obesity	194,937 (31.5)	23,466 (31.1)	21,314 (27.5)
Liver Cirrhosis	8,940 (1.4)	782 (1.0)	1,680 (2.2)
**Drug Initiation Over Study Period, n (%)**			
SGLT2i	119,627 (17.1)	11,529 (13.7)	14,803 (16.7)
GLP1-RA	114,942 (16.4)	12,175 (14.5)	12,325 (13.9)
DPP4i	111,887 (16.0)	18,830 (22.4)	21,361 (24.1)
Sulfonylurea	273,369 (39.1)	33,036 (39.2)	29,026 (32.7)
**Stratification by HF Status**			
	**White (n=113,728)**	**Black (n=16,076)**	**Hispanic (n=13,116)**
**Age, mean (SD), years**	77.0 (7.5)	75.3 (7.4)	76.5 (7.4)
**Male, n (%)**	57,460 (50.5)	5,602 (34.9)	5,526 (42.1)
**SVI Percentile**			
SVI (Overall), median (IQR)	53.6 (42.1)	70.2 (30.5)	76.8 (34.2)
**Baseline Complications, n (%)**			
Diabetic Neuropathy	40,920 (36.0)	6,195 (38.5)	5,325 (40.6)
Diabetic Retinopathy	16,009 (14.1)	3,186 (19.8)	3,217 (24.5)
Diabetic Nephropathy	14,804 (13.0)	2,758 (17.2)	2,791 (21.3)
**Baseline Comorbid Medical Conditions, n (%)**			
CKD	40,963 (36.0)	6,758 (42.0)	5,027 (38.3)
ASCVD	93,111 (81.9)	12,616 (78.5)	10,656 (81.2)
Hypertension	111,186 (97.8)	15,969 (99.3)	12,963 (98.8)
Hyperlipidemia	102,978 (90.6)	14,168 (88.1)	11,972 (91.3)
Atrial Fibrillation	53,448 (47.8)	4,400 (27.4)	4,012 (30.6)
Obesity	47,890 (42.1)	6,766 (42.1)	5,062 (38.6)
Liver Cirrhosis	2,746 (2.4)	297 (1.9)	485 (3.7)
**Drug Initiation Over Study Period, n (%)**			
SGLT2i	17,163 (15.1)	1,805 (11.2)	2,043 (15.6)
GLP1-RA	20,090 (17.7)	2,554 (15.9)	2,226 (17.0)
DPP4i	25,637 (22.5)	4,545 (28.3)	4,054 (30.9)

Sulfonylurea	50,838 (44.7)	7,172 (44.6)	4,793 (36.5)
**Stratification by ASCVD Status**			
	**White (n=310,924)**	**Black (n=35,954)**	**Hispanic (n=36,594)**
**Age, mean (SD), years**	75.5 (6.9)	74.7 (7.0)	75.3 (6.9)
**Male, n (%)**	172,770 (55.6)	13,272 (36.9)	15,935 (43.6)
**SVI Percentile**			
SVI (Overall), median (IQR)	52.4 (42.1)	68.6 (32.0)	76.8 (34.5)
**Baseline Complications, n (%)**			
Diabetic Neuropathy	98,134 (31.6)	12,919 (35.9)	13,946 (38.1)
Diabetic Retinopathy	41,333 (13.3)	6,817 (19.0)	8,354 (23.8)
Diabetic Nephropathy	30,166 (9.7)	4,750 (13.2)	5,522 (15.1)
**Baseline Comorbid Medical Conditions, n (%)**			
CKD	72,874 (23.4)	10,888 (30.3)	9,185 (25.1)
HF	93,111 (30.0)	12,616 (35.1)	10,656 (29.1)
Hypertension	298,869 (96.1)	35,458 (98.6)	35,647 (97.4)
Hyperlipidemia	286,836 (92.3)	31,975 (88.9)	33,371 (91.2)
Atrial Fibrillation	83,560 (26.9)	5,778 (16.1)	5,907 (16.1)
Obesity	107,569 (34.6)	12,339 (34.3)	11,632 (31.8)
Liver Cirrhosis	5,117 (1.7)	481 (1.3)	935 (2.6)
**Drug Initiation Over Study Period, n (%)**			
SGLT2i	58,263 (18.7)	5,013 (13.9)	6,750 (18.5)
GLP1-RA	56,374 (18.1)	5,758 (16.0)	6,109 (16.7)
DPP4i	61,727 (19.9)	9,626 (26.8)	10,849 (29.7)
Sulfonylurea	134,550 (43.3)	15,557 (43.3)	12,886 (35.2)
**Stratification by CKD status**			
	**White (n=111,090)**	**Black (n=17,583)**	**Hispanic (n=14,232)**
**Age, mean (SD), years**	76.5 (7.1)	75.1 (7.0)	76.2 (7.2)
**Male, n (%)**	57,055 (51.4)	6,764 (38.5)	6,350 (44.6)
**SVI Percentile**			
SVI (Overall), median (IQR)	51.7 (41.1)	69.1 (31.5)	76.8 (36.6)
**Baseline Complications, n (%)**			
Diabetic Neuropathy	39,495 (35.6)	6,509 (37.0)	5,794 (40.7)
Diabetic Retinopathy	17,295 (15.6)	4,008 (22.8)	4,145 (29.1)
Diabetic Nephropathy	27,987 (25.2)	5,166 (29.4)	5,360 (37.7)
**Baseline Comorbid Medical Conditions, n (%)**			
ASCVD	72,874 (65.6)	10,888 (61.9)	9,185 (64.5)
HF	40,963 (36.9)	6,758 (38.4)	5,027 (35.3)
Hypertension	108,776 (97.9)	17,432 (99.1)	14,048 (98.7)
Hyperlipidemia	101,690 (91.5)	15,539 (88.4)	12,949 (91.0)
Atrial Fibrillation	32,416 (29.2)	2,972 (16.9)	2,477 (17.4)
Obesity	43,838 (39.5)	6,646 (37.8)	4,909 (34.5)
Liver Cirrhosis	2,146 (1.9)	299 (1.7)	454 (3.2)
**Drug Initiation Over Study Period, n (%)**			
SGLT2i	13,368 (12.0)	1,598 (9.1)	1,718 (12.1)
GLP1-RA	24,182 (21.8)	3,351 (19.1)	2,853 (20.1)
DPP4i	27,300 (24.6)	5,523 (31.4)	4,883 (34.3)
Sulfonylurea	46,240 (41.6)	7,111 (40.4)	4,778 (33.6)
**Stratification by No Cardiorenal Conditions**			
	**White (n=256,036)**	**Black (n=30,618)**	**Hispanic (n=34,144)**
**Age, mean (SD), years**	72.8 (5.8)	72.3 (5.8)	
**Male, n (%)**	57,055 (51.4)	9,672 (31.6)	13,368 (39.2)

**SVI Percentile**			
SVI (Overall), median (IQR)	50.4 (41.4)	67.6 (34.2)	
**Baseline Complications, n (%)**			
Diabetic Neuropathy	46,149 (18.0)	5,795 (18.9)	6,804 (19.9)
Diabetic Retinopathy	24,498 (9.6)	4,235 (13.8)	5,405 (15.9)
Diabetic Nephropathy	8,528 (3.3)	1,206 (3.9)	1,526 (4.5)
**Baseline Comorbid Medical Conditions, n (%)**			
Hypertension	220,834 (86.3)	28,684 (93.7)	29,654 (86.9)
Hyperlipidemia	211,592 (82.6)	24,097 (78.7)	26,829 (78.6)
Atrial Fibrillation	17,338 (6.8)	865 (2.8)	970 (2.8)
Obesity	67,966 (26.6)	7,902 (25.8)	7,630 (22.4)
Liver Cirrhosis	2,934 (1.2)	200 (0.7)	548 (1.6)
**Drug Initiation Over Study Period, n (%)**			
SGLT2i	54,197 (21.2)	5,511 (18.0)	7,128 (20.9)
GLP1-RA	46,806 (18.3)	4,669 (15.3)	4,907 (14.8)
DPP4i	39,152 (15.3)	6,702 (21.9)	8,450 (24.8)
Sulfonylurea	115,881 (45.3)	13,736 (44.9)	13,659 (40.0)
T2DM, Type 2 diabetes mellitus; HF, heart failure; CKD, chronic kidney disease; ASCVD, atherosclerotic cardiovascular disease; SVI, social vulnerability index; SVI-MH, social vulnerability index-minority health.			

ESM Table 2. Baseline clinical characteristics for T2DM patients initiating 2nd-line glucose-lowering therapies by HF, CKD, ASCVD, 2013-2019

	All (n=838,361)	HF (n= 152,214)	ASCVD (n= 412,890)	CKD (n= 154,849)	No cardiorenal conditions (n= 350,707)
**Age, mean (SD), years**	74.3 (6.6)	76.7 (7.5)	75.4 (6.9)	76.4 (7.1)	72.6 (5.8)
**Male, n (%)**	396,542 (47.3)	73,401 (48.2)	218,418 (52.9)	76,654 (49.5)	147,655 (42.1)
**Race, n (%)**					
White	619,825 (73.9)	113,728 (74.7)	310,924 (75.3)	111,090 (71.7)	256,036 (73.0)
Black	75,570 (9.0)	16,076 (10.6)	35,954 (8.7)	17,583 (11.4)	30,618 (8.7)
Hispanic	77,515 (9.3)	13,116 (8.6)	36,594 (8.9)	14,232 (9.2)	34,144 (9.7)
Other	65,451 (7.8)	9,294 (6.1)	29,418 (7.1)	11,944 (7.7)	29,909 (8.5)
**SVI Percentile**					
SVI (Overall), median (IQR)	55.3 (42.5)	57.4 (41.8)	60.0 (43.3)	55.9 (42.5)	53.7 (42.2)
**Complications, n (%)**					
Diabetic Neuropathy	217,169 (25.9)	55,572 (36.5)	133,824 (32.4)	55,489 (35.8)	63,333 (18.1)
Diabetic Retinopathy	111,125 (13.3)	24,343 (16.0)	62,216 (15.1)	28,086 (18.1)	38,108 (10.9)
Diabetic Nephropathy	72,523 (8.7)	22,194 (14.6)	44,562 (10.8)	42,648 (27.5)	12,852 (3.7)
**Baseline Medications, n (%)**					
Metformin	561,086 (66.9)	82,284 (54.1)	259,223 (62.8)	70,430 (45.5)	261,881 (74.7)
Insulin	182,131 (21.7)	46,469 (30.5)	104,722 (25.4)	48,875 (31.6)	58,053 (16.6)
Other glucose-lowering therapies	24,499 (2.9)	5,294 (3.5)	14,144 (3.4)	6,373 (4.1)	7,971 (2.3)
**Baseline Comorbid Medical Conditions, n (%)**					
CKD	154,849 (18.5)	56,294 (37.0)	100,023 (24.2)	154,849 (100)	0 (0)
ASCVD	412,890 (49.3)	123,817 (81.3)	412,890 (100)	100,023 (64.6)	0 (0)
HF	152,214 (18.2)	152,214 (100)	123,817 (30.0)	56,294 (36.4)	0 (0)
Hypertension	775,195 (92.5)	149,213 (98.0)	398,242 (96.5)	151,971 (98.1)	305,030 (87.0)
Hyperlipidemia	729,982 (87.1)	137,625 (90.4)	379,188 (91.8)	141,132 (91.1)	287,325 (81.9)
Atrial Fibrillation	134,215 (16.0)	64,944 (42.7)	100,335 (24.3)	40,041 (25.9)	20,259 (5.8)
Obesity	250,854 (29.9)	62,116 (40.8)	137,276 (33.2)	57,939 (37.4)	87,725 (25.0)
Liver Cirrhosis	12,358 (1.5)	3,759 (2.5)	7,005 (1.7)	3,147 (2.0)	4,054 (1.2)

Abbreviations: ASCVD, atherosclerotic cardiovascular disease; CKD, chronic kidney disease; HF, heart failure; T2DM, type 2 diabetes mellitus; SVI, social vulnerability index

ESM Table 3. Proportion of new initiators of SGLT2i by year, race/ethnicity, and conditions

Heart Failure			
**Year**	**White**	**Black**	**Hispanic**
2013	2.0	1.0	1.8
2014	10.4	6.6	9.1
2015	14.2	9.9	15.1
2016	13.2	9.6	14.5
2017	16.1	12.7	18.1
2018	16.7	12.5	18.4
2019	25.0	21.5	26.6
**ASCVD**			
**Year**	**White**	**Black**	**Hispanic**
2013	2.7	1.7	2.0
2014	13.6	9.4	12.0
2015	19.7	14.1	19.5
2016	18.0	13.4	19.1
2017	20.9	17.0	22.2
2018	20.5	15.1	21.4
2019	27.9	23.1	28.8
**CKD**			
**Year**	**White**	**Black**	**Hispanic**
2013	1.9	1.0	1.3
2014	7.9	5.7	8.3
2015	10.7	7.4	10.3
2016	9.6	7.0	9.1
2017	11.8	9.4	12.7
2018	12.2	9.4	13.1
2019	19.6	16.8	20.7
**None**			
**Year**	**White**	**Black**	**Hispanic**
2013	2.8	1.8	1.9
2014	14.5	10.2	12.5
2015	21.4	16.1	20.2
2016	19.7	15.1	20.4
2017	21.6	18.4	22.8
2018	20.6	16.9	22.0
2019	26.7	23.8	28.6

ESM Table 4. Proportion of new initiators of GLP1-RA by year, race/ethnicity, and conditions

Heart Failure			
**Year**	**White**	**Black**	**Hispanic**
2013	10.5	8.2	11.3
2014	10.5	9.5	11.3
2015	11.4	9.8	12.0
2016	15.2	12.8	12.7
2017	18.7	17.2	17.9
2018	23.8	22.4	24.2
2019	26.0	26.1	24.6
**ASCVD**			
**Year**	**White**	**Black**	**Hispanic**
2013	11.6	7.8	11.4
2014	10.8	9.2	10.2
2015	12.3	10.5	11.6
2016	16.0	13.5	13.7
2017	19.4	17.7	18.3
2018	24.5	23.5	22.9
2019	26.1	26.3	25.3
**CKD**			
**Year**	**White**	**Black**	**Hispanic**
2013	11.9	8.4	11.8
2014	12.5	10.1	11.9
2015	14.5	11.8	13.0
2016	18.4	15.5	16.7
2017	22.0	20.4	21.9
2018	27.7	25.6	25.7
2019	30.1	29.7	28.2
**None**			
**Year**	**White**	**Black**	**Hispanic**
2013	12.5	8.6	10.4
2014	11.6	8.9	9.7
2015	12.8	10.5	10.6
2016	16.3	13.7	13.0
2017	19.5	17.7	17.0
2018	24.7	23.1	22.0
2019	26.6	26.5	24.8

ESM Table 5. Proportion of new initiators of DPP4i by year, race/ethnicity, and conditions

Heart Failure			
Year	White	Black	Hispanic
2013	23.2	24.4	35.0
2014	23.5	25.8	31.1
2015	27.3	31.6	35.1
2016	25.9	33.4	37.0
2017	23.6	30.3	30.4
2018	21.3	31.2	28.0
2019	16.5	22.4	23.3
ASCVD			
Year	White	Black	Hispanic
2013	23.4	25.7	35.8
2014	22.9	27.0	32.9
2015	23.9	30.0	34.0
2016	22.1	31.4	34.3
2017	19.4	27.4	27.6
2018	17.5	26.0	26.0
2019	13.6	20.9	20.1
CKD			
Year	White	Black	Hispanic
2013	26.9	29.2	39.2
2014	27.2	30.5	34.0
2015	30.9	36.4	40.7
2016	28.0	36.0	39.4
2017	25.8	33.5	35.3
2018	22.8	31.5	32.6
2019	17.9	25.0	25.8
None			
Year	White	Black	Hispanic
2013	22.3	26.1	33.4
2014	21.3	26.2	30.1
2015	21.9	28.7	31.7
2016	20.0	28.8	31.3
2017	17.3	24.8	26.5
2018	15.5	23.1	24.3
2019	11.9	18.0	18.7

ESM Table 6. Proportion of new initiators of SU by year, race/ethnicity, and conditions

Heart Failure			
**Year**	**White**	**Black**	**Hispanic**
2013	64.3	66.4	51.9
2014	55.6	58.1	48.5
2015	47.0	48.7	37.8
2016	45.7	44.2	35.8
2017	41.7	39.8	33.6
2018	38.3	33.9	29.4
2019	32.5	30.0	25.5
**ASCVD**			
**Year**	**White**	**Black**	**Hispanic**
2013	62.2	64.9	50.8
2014	52.7	54.4	44.9
2015	44.1	45.5	34.8
2016	43.9	41.7	32.9
2017	40.4	37.9	31.8
2018	37.5	35.4	29.7
2019	32.4	29.7	25.8
**CKD**			
**Year**	**White**	**Black**	**Hispanic**
2013	59.3	61.3	47.8
2014	52.5	53.8	45.9
2015	43.9	44.4	36.0
2016	44.0	41.5	34.8
2017	40.3	36.8	30.2
2018	37.3	33.5	28.5
2019	32.4	28.6	25.3
**None**			
**Year**	**White**	**Black**	**Hispanic**
2013	62.3	63.6	54.4
2014	52.7	54.7	47.7
2015	44.0	44.7	37.5
2016	44.1	42.4	35.2
2017	41.5	39.1	33.6
2018	39.2	37.0	31.6
2019	34.7	31.7	27.8

ESM Table 7. Fully Adjusted Odds Ratios of likelihood of starting 2nd line glucose-lowering therapies by class overall in Black and Hispanic vs White patients (2013-2019) (sulfonylurea=reference)

	HF					
**Race**	**GLP1-RA**		**SGLT2i**		**DPP-4i**	
	**OR**	**95% CI**	**OR**	**95% CI**	**OR**	**95% CI**
Black	0.67	0.63-0.70	0.65	0.61-0.68	1.04	0.99-1.08
Hispanic	0.89	0.84-0.94	0.99	0.93-1.05	1.25	1.19-1.31
	**ASCVD**					
	**OR**	**95% CI**	**OR**	**95% CI**	**OR**	**95% CI**
Black	0.67	0.65-0.70	0.67	0.64-0.69	1.10	1.06-1.13
Hispanic	0.84	0.81-0.87	0.94	0.91-0.98	1.36	1.32-1.40
	**CKD**					
	**OR**	**95% CI**	**OR**	**95% CI**	**OR**	**95% CI**
Black	0.70	0.66-0.73	0.68	0.64-0.72	1.12	1.08-1.17
Hispanic	0.84	0.80-0.89	0.95	0.89-1.02	1.32	1.26-1.38
	**None**					
	**OR**	**95% CI**	**OR**	**95% CI**	**OR**	**95% CI**
Black	0.71	0.68-0.73	0.76	0.74-0.79	1.52	1.47-1.57
Hispanic	0.75	0.72-0.78	0.93	0.90-0.96	0.93	0.90-0.96

ESM Table 8. Fully Adjusted Odds Ratios of likelihood of starting 2nd line glucose-lowering therapies by class in Black vs White patients adjusted for age, sex, conditions, medications, and SVI by year (sulfonylurea=reference)

	Heart failure					
Year	GLP1-RA		SGLT2i		DPP-4i	
	OR	95% CI	OR	95% CI	OR	95% CI
2013	0.53	0.44-0.63	0.40	0.25-0.63	0.84	0.74-0.94
2014	0.65	0.54-0.77	0.49	0.40-0.60	0.87	0.77-0.98
2015	0.55	0.46-0.65	0.51	0.43-0.60	0.91	0.81-1.01
2016	0.67	0.57-0.78	0.62	0.52-0.73	1.12	1.00-1.25
2017	0.69	0.60-0.79	0.71	0.61-0.83	1.10	0.98-1.23
2018	0.74	0.65-0.84	0.71	0.61-0.83	1.34	1.20-1.50
2019	0.81	0.73-0.91	0.83	0.74-0.94	1.22	1.08-1.36
	ASCVD					
Year	GLP1-RA		SGLT2i		DPP-4i	
	OR	95% CI	OR	95% CI	OR	95% CI
2013	0.50	0.44-0.56	0.49	0.38-0.62	0.87	0.80-0.94
2014	0.63	0.56-0.70	0.56	0.50-0.62	0.95	0.89-1.03
2015	0.60	0.54-0.67	0.59	0.54-0.65	1.01	0.94-1.08
2016	0.67	0.61-0.74	0.68	0.62-0.75	1.23	1.14-1.32
2017	0.72	0.66-0.79	0.78	0.72-0.85	1.19	1.10-1.28
2018	0.72	0.67-0.79	0.68	0.62-0.74	1.22	1.13-1.32
2019	0.84	0.78-0.91	0.82	0.76-0.89	1.31	1.21-1.43
	CKD					
Year	GLP1-RA		SGLT2i		DPP-4i	
	OR	95% CI	OR	95% CI	OR	95% CI
2013	0.54	0.44-0.66	0.48	0.28-0.80	0.90	0.79-1.02
2014	0.62	0.52-0.73	0.59	0.47-0.73	0.93	0.83-1.05
2015	0.59	0.50-0.69	0.55	0.46-0.67	1.03	0.93-1.15
2016	0.67	0.59-0.77	0.67	0.56-0.80	1.17	1.06-1.30
2017	0.74	0.65-0.84	0.74	0.63-0.87	1.19	1.07-1.32
2018	0.75	0.67-0.85	0.70	0.60-0.82	1.26	1.13-1.40
2019	0.84	0.76-0.93	0.83	0.74-0.93	1.35	1.22-1.49
	None					
Year	GLP1-RA		SGLT2i		DPP-4i	
	OR	95% CI	OR	95% CI	OR	95% CI
2013	0.58	0.51-0.66	0.48	0.28-0.80	1.12	1.03-1.22
2014	0.54	0.48-0.62	0.61	0.55-0.68	1.08	0.99-1.17
2015	0.64	0.57-0.71	0.69	0.64-0.76	1.20	1.11-1.30
2016	0.70	0.63-0.77	0.73	0.67-0.79	1.37	1.26-1.48
2017	0.75	0.68-0.82	0.83	0.76-0.90	1.32	1.21-1.45
2018	0.76	0.70-0.84	0.82	0.75-0.90	1.28	1.17-1.41
2019	0.87	0.80-0.94	0.93	0.86-1.01	1.36	1.23-1.50

ESM Table 9. Sensitivity Analysis Odds Ratios of likelihood of starting 2nd line glucose-lowering therapies by class overall in Black and Hispanic vs White patients (2013-2019) (sulfonylurea=reference)

	HF					
**Race**	**GLP1-RA**		**SGLT2i**		**DPP-4i**	
	**OR**	**95% CI**	**OR**	**95% CI**	**OR**	**95% CI**
Black	0.90	0.86-0.95	0.75	0.71-0.79	1.26	1.21-1.31
Hispanic	1.17	1.12-1.24	1.26	1.20-1.33	1.68	1.61-1.75
	**ASCVD**					
	**OR**	**95% CI**	**OR**	**95% CI**	**OR**	**95% CI**
Black	0.88	0.86-0.91	0.74	0.72-0.77	1.35	1.31-1.39
Hispanic	1.13	1.10-1.17	1.21	1.17-1.25	1.84	1.79-1.89
	**CKD**					
	**OR**	**95% CI**	**OR**	**95% CI**	**OR**	**95% CI**
Black	0.90	0.86-0.94	0.78	0.73-0.82	1.32	1.27-1.37
Hispanic	1.14	1.09-1.20	1.24	1.12-1.32	1.73	1.66-1.81
	**None**					
	**OR**	**95% CI**	**OR**	**95% CI**	**OR**	**95% CI**
Black	0.84	0.81-0.87	0.86	0.83-0.89	1.44	1.40-1.49
Hispanic	0.89	0.86-0.92	1.12	1.08-1.15	1.83	1.78-1.89

ESM Table 10. Sensitivity Analysis Odds Ratios of likelihood of starting 2nd line glucose-lowering therapies by class in Black vs White patients by year (sulfonylurea=reference)

	Heart failure					
Year	GLP1-RA		SGLT2i		DPP-4i	
	OR	95% CI	OR	95% CI	OR	95% CI
2013	0.61	0.51-0.73	0.46	0.29-0.72	0.92	0.82-1.03
2014	0.73	0.62-0.86	0.51	0.42-0.62	0.98	0.88-1.10
2015	0.67	0.57-0.79	0.56	0.48-0.66	1.05	0.95-1.17
2016	0.74	0.64-0.86	0.64	0.55-0.76	1.23	1.11-1.37
2017	0.82	0.72-0.93	0.74	0.64-0.86	1.30	1.16-1.45
2018	0.91	0.81-1.02	0.77	0.67-0.88	1.63	1.46-1.82
2019	0.99	0.89-1.09	0.89	0.80-1.00	1.47	1.32-1.64
	ASCVD					
Year	GLP1-RA		SGLT2i		DPP-4i	
	OR	95% CI	OR	95% CI	OR	95% CI
2013	0.55	0.49-0.62	0.56	0.44-0.71	0.94	0.88-1.02
2014	0.72	0.65-0.81	0.61	0.55-0.68	1.07	0.99-1.15
2015	0.72	0.65-0.80	0.63	0.57-0.69	1.14	1.06-1.22
2016	0.78	0.72-0.86	0.71	0.65-0.78	1.38	1.29-1.49
2017	0.88	0.81-0.96	0.83	0.76-0.90	1.40	1.30-1.51
2018	0.92	0.85-0.99	0.74	0.68-0.81	1.50	1.39-1.61
2019	1.04	0.97-1.12	0.91	0.84-0.98	1.62	1.50-1.75
	CKD					
Year	GLP1-RA		SGLT2i		DPP-4i	
	OR	95% CI	OR	95% CI	OR	95% CI
2013	0.57	0.47-0.70	0.48	0.29-0.81	0.95	0.84-1.07
2014	0.66	0.56-0.78	0.60	0.48-0.74	1.01	0.91-1.13
2015	0.68	0.58-0.79	0.58	0.49-0.69	1.13	1.02-1.25
2016	0.76	0.67-0.86	0.68	0.57-0.81	1.28	1.16-1.41
2017	0.87	0.77-0.98	0.78	0.67-0.91	1.35	1.22-1.50
2018	0.92	0.82-1.02	0.77	0.67-0.89	1.48	1.34-1.64
2019	1.00	0.91-1.10	0.90	0.81-1.01	1.55	1.41-1.71
	None					
Year	GLP1-RA		SGLT2i		DPP-4i	
	OR	95% CI	OR	95% CI	OR	95% CI
2013	0.60	0.55-0.65	0.62	0.53-0.74	1.04	0.99-1.10
2014	0.68	0.63-0.73	0.64	0.59-0.68	1.11	1.06-1.17
2015	0.73	0.68-0.78	0.68	0.65-0.72	1.21	1.15-1.27
2016	0.79	0.74-0.84	0.74	0.69-0.78	1.40	1.34-1.47
2017	0.88	0.83-0.94	0.85	0.81-0.90	1.44	1.37-1.52
2018	0.92	0.87-0.97	0.83	0.78-0.88	1.51	1.43-1.59
2019	1.03	0.98-1.09	0.96	0.92-1.01	1.61	1.52-1.70

ESM Table 11. Sensitivity Analysis Odds Ratios of likelihood of starting 2nd line glucose-lowering therapies by class overall in Black and Hispanic vs White patients restricting to first initiation episode (2013-2019) (sulfonylurea=reference)

	**HF**					
**Race**	**GLP1-RA**		**SGLT2i**		**DPP-4i**	
	**OR**	**95% CI**	**OR**	**95% CI**	**OR**	**95% CI**
Black	0.65	0.61-0.69	0.65	0.61-0.69	1.04	0.99-1.08
Hispanic	0.88	0.83-0.95	0.94	0.87-1.00	1.26	1.20-1.33
	**ASCVD**					
	**OR**	**95% CI**	**OR**	**95% CI**	**OR**	**95% CI**
Black	0.65	0.63-0.70	0.67	0.64-0.69	1.09	1.06-1.13
Hispanic	0.82	0.78-0.85	0.90	0.87-0.94	1.37	1.33-1.41
	**CKD**					
	**OR**	**95% CI**	**OR**	**95% CI**	**OR**	**95% CI**
Black	0.68	0.65-0.72	0.69	0.65-0.74	1.13	1.08-1.18
Hispanic	0.80	0.75-0.85	0.89	0.83-0.96	1.32	1.25-1.39
	**None**					
	**OR**	**95% CI**	**OR**	**95% CI**	**OR**	**95% CI**
Black	0.70	0.67-0.73	0.76	0.74-0.79	1.26	1.22-1.31
Hispanic	0.72	0.69-0.75	0.89	0.85-0.92	1.51	1.46-1.57

ESM Table 12. Fully Adjusted Odds Ratios of likelihood of starting 2nd line glucose-lowering therapies by class in Hispanic vs White patients adjusted for age, sex, conditions, medications, and SVI by year (sulfonylurea=reference)

	Heart failure					
Year	GLP1-RA		SGLT2i		DPP-4i	
	OR	95% CI	OR	95% CI	OR	95% CI
2013	0.90	0.74-1.08	0.74	0.49-1.10	1.31	1.16-1.48
2014	0.89	0.73-1.08	0.67	0.55-0.83	1.10	0.96-1.26
2015	0.87	0.72-1.05	0.86	0.73-1.02	1.18	1.03-1.34
2016	0.79	0.66-0.94	1.00	0.84-1.18	1.36	1.20-1.54
2017	0.91	0.78-1.07	1.15	0.98-1.34	1.19	1.04-1.36
2018	0.95	0.82-1.10	1.12	0.97-1.31	1.24	1.08-1.42
2019	0.96	0.84-1.09	1.13	0.99-1.28	1.41	1.24-1.61
	ASCVD					
Year	GLP1-RA		SGLT2i		DPP-4i	
	OR	95% CI	OR	95% CI	OR	95% CI
2013	0.84	0.75-0.94	0.58	0.46-0.73	1.34	1.24-1.44
2014	0.77	0.68-0.87	0.68	0.61-0.76	1.23	1.14-1.33
2015	0.85	0.76-0.94	0.91	0.83-1.00	1.37	1.27-1.48
2016	0.85	0.76-0.94	1.06	0.97-1.16	1.57	1.45-1.70
2017	0.87	0.79-0.95	1.08	0.99-1.18	1.33	1.23-1.45
2018	0.85	0.78-0.93	1.03	0.95-1.12	1.34	1.24-1.46
2019	0.92	0.85-0.99	1.04	0.97-1.13	1.38	1.27-1.50
	CKD					
Year	GLP1-RA		SGLT2i		DPP-4i	
	OR	95% CI	OR	95% CI	OR	95% CI
2013	0.83	0.67-1.03	0.54	0.31-0.92	1.33	1.16-1.53
2014	0.75	0.61-0.91	0.78	0.62-0.98	1.03	0.90-1.18
2015	0.77	0.64-0.92	0.81	0.66-0.99	1.28	1.13-1.46
2016	0.80	0.68-0.94	0.90	0.74-1.09	1.36	1.20-1.53
2017	0.94	0.81-1.08	1.09	0.93-1.29	1.40	1.24-1.58
2018	0.87	0.76-0.99	1.04	0.89-1.22	1.36	1.21-1.53
2019	0.89	0.79-1.00	1.04	0.92-1.18	1.44	1.28-1.62
	None					
Year	GLP1-RA		SGLT2i		DPP-4i	
	OR	95% CI	OR	95% CI	OR	95% CI
2013	0.61	0.54-0.70	0.56	0.44-0.72	1.31	1.21-1.43
2014	0.66	0.58-0.75	0.71	0.64-0.79	1.21	1.11-1.31
2015	0.68	0.61-0.77	0.83	0.76-0.90	1.40	1.29-1.52
2016	0.75	0.68-0.83	1.04	0.96-1.13	1.81	1.67-1.96
2017	0.72	0.65-0.80	1.02	0.94-1.11	1.68	1.54-1.83
2018	0.83	0.76-0.91	1.10	1.01-1.20	1.72	1.57-1.88
2019	0.89	0.81-0.96	1.10	1.02-1.19	1.76	1.60-1.94

ESM Table 13. Sensitivity Analysis Odds Ratios of likelihood of starting 2nd line glucose-lowering therapies by class in Hispanic vs White patients by year (sulfonylurea=reference)

	Heart failure					
Year	GLP1-RA		SGLT2i		DPP-4i	
	OR	95% CI	OR	95% CI	OR	95% CI
2013	1.11	0.94-1.32	1.02	0.69-1.51	1.61	1.43-1.90
2014	1.10	0.92-1.33	0.85	0.70-1.04	1.35	1.19-1.53
2015	1.17	0.99-1.39	1.15	0.98-1.35	1.45	1.28-1.63
2016	0.98	0.84-1.16	1.24	1.06-1.45	1.64	1.46-1.85
2017	1.12	0.97-1.30	1.33	1.15-1.54	1.50	1.32-1.70
2018	1.25	1.10-1.42	1.38	1.20-1.59	1.64	1.45-1.86
2019	1.16	1.03-1.31	1.34	1.19-1.51	1.76	1.56-1.99
	ASCVD					
Year	GLP1-RA		SGLT2i		DPP-4i	
	OR	95% CI	OR	95% CI	OR	95% CI
2013	1.06	0.95-1.17	0.87	0.70-1.09	1.60	1.49-1.72
2014	1.02	0.91-1.14	0.94	0.85-1.04	1.52	1.41-1.63
2015	1.09	0.99-1.21	1.16	1.06-1.26	1.63	1.51-1.75
2016	1.07	0.97-1.18	1.31	1.20-1.43	1.85	1.72-2.00
2017	1.12	1.03-1.22	1.28	1.18-1.39	1.64	1.52-1.77
2018	1.11	1.03-1.21	1.26	1.16-1.37	1.72	1.59-1.86
2019	1.19	1.10-1.28	1.30	1.21-1.40	1.74	1.61-1.89
	CKD					
Year	GLP1-RA		SGLT2i		DPP-4i	
	OR	95% CI	OR	95% CI	OR	95% CI
2013	1.10	0.91-1.33	0.76	0.46-1.26	1.58	1.39-1.80
2014	0.96	0.80-1.16	1.05	0.84-1.30	1.29	1.14-1.47
2015	0.98	0.83-1.16	1.01	0.84-1.21	1.51	1.34-1.71
2016	1.04	0.90-1.20	1.08	0.91-1.30	1.62	1.45-1.81
2017	1.20	1.06-1.37	1.30	1.11-1.51	1.70	1.52-1.91
2018	1.15	1.02-1.29	1.29	1.12-1.50	1.73	1.55-1.94
2019	1.14	1.03-1.27	1.28	1.14-1.43	1.78	1.59-1.98
	None					
Year	GLP1-RA		SGLT2i		DPP-4i	
	OR	95% CI	OR	95% CI	OR	95% CI
2013	0.87	0.81-0.94	0.78	0.67-0.92	1.51	1.44-1.59
2014	0.89	0.82-0.96	0.91	0.85-0.98	1.43	1.35-1.50
2015	0.91	0.84-0.97	1.04	0.99-1.10	1.55	1.47-1.63
2016	0.94	0.88-1.01	1.22	1.15-1.29	1.80	1.71-1.89
2017	1.01	0.95-1.07	1.22	1.16-1.29	1.75	1.65-1.84
2018	1.05	1.00-1.11	1.27	1.20-1.34	1.80	1.71-1.91
2019	1.13	1.07-1.19	1.31	1.25-1.37	1.85	1.75-1.96

ESM Figure 1: Flow Diagram

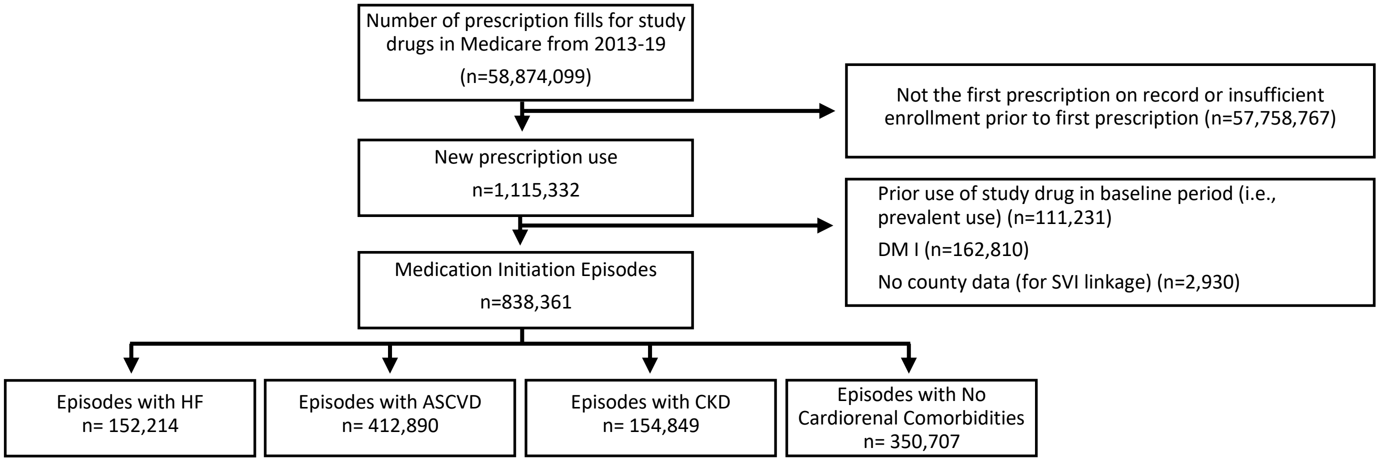

ESM Figure 2: Temporal patterns in proportion of new initiators of DPP4i by year, race/ethnicity, and conditions

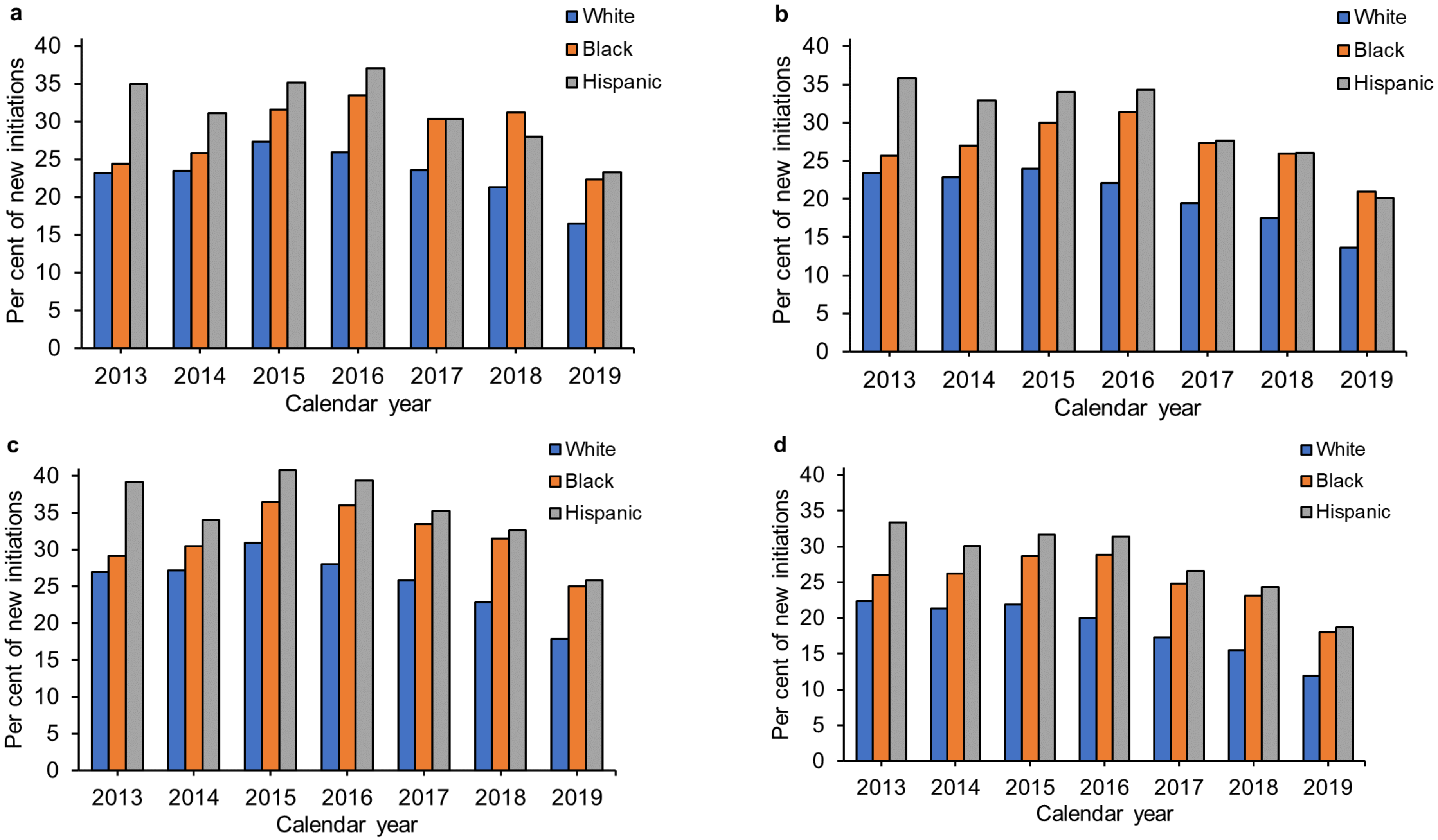

ESM Figure 2: New initiators of DPP4i by comorbid cardiorenal status (A: comorbid HF; B: comorbid ASCVD; C: comorbid CKD) and among patients without comorbid cardiorenal conditions (D) stratified by year and race. The figures describe the proportion of new initiators of DPP4i between 2013-2019 for White, Black, and Hispanic patients.

ESM Figure 3: Temporal patterns in proportion of new initiators of SU by year, race/ethnicity, and conditions

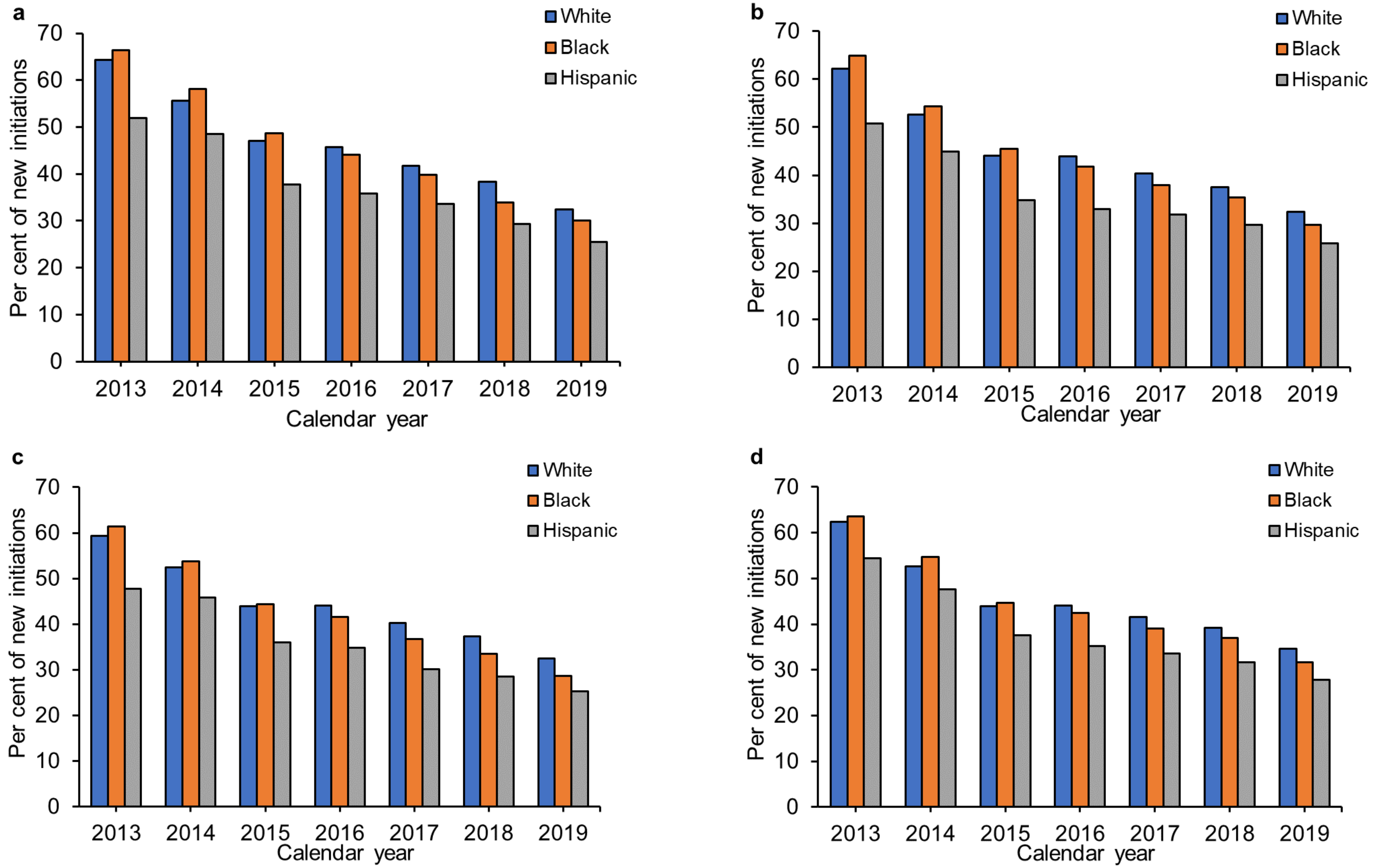

ESM Figure 3: New initiators of SU by comorbid cardiorenal status (A: comorbid HF; B: comorbid ASCVD; C: comorbid CKD) and among patients without comorbid cardiorenal conditions (D) stratified by year and race. The figures describe the proportion of new initiators of SUs between 2013-2019 for White, Black, and Hispanic patients.
